# Supplementary material for: Unveiling the Bioactive Potential of the Invasive Jellyfish Phyllorhiza punctata Through Integrative Transcriptomic and Proteomic Analyses
Source: Biomolecules. 2025 Aug 4;15(8):1121. doi: 10.3390/biom15081121 (PMC12383608; doi:10.3390/biom15081121)
Supplement: Supplementary file 1 [file biomolecules-15-01121-s001.zip › Suplementary Material/FileS3/PEPFOLD4/XP_065066428_8507/clusters.html]

 PEP-FOLD clusters


Download archive of all models  here   

|  |  |  |
| --- | --- | --- |
| # | file | sOPEP |
| # cluster 1 - Size: 5 |  |  |
|  | XP\_065066428\_8507-model1 | -24.0299 |
|  | XP\_065066428\_8507-model1.1 | -21.9165 |
|  | XP\_065066428\_8507-model1.2 | -19.8039 |
|  | XP\_065066428\_8507-model1.3 | -16.3211 |
|  | XP\_065066428\_8507-model1.4 | -15.3378 |
| # cluster 2 - Size: 3 |  |  |
|  | XP\_065066428\_8507-model2 | -23.7715 |
|  | XP\_065066428\_8507-model2.1 | -22.1969 |
|  | XP\_065066428\_8507-model2.2 | -17.5826 |
| # cluster 3 - Size: 4 |  |  |
|  | XP\_065066428\_8507-model3 | -23.7671 |
|  | XP\_065066428\_8507-model3.1 | -22.7808 |
|  | XP\_065066428\_8507-model3.2 | -22.715 |
|  | XP\_065066428\_8507-model3.3 | -21.5058 |
| # cluster 4 - Size: 3 |  |  |
|  | XP\_065066428\_8507-model4 | -23.6671 |
|  | XP\_065066428\_8507-model4.1 | -19.7924 |
|  | XP\_065066428\_8507-model4.2 | -15.8094 |
| # cluster 5 - Size: 4 |  |  |
|  | XP\_065066428\_8507-model5 | -21.9398 |
|  | XP\_065066428\_8507-model5.1 | -19.2057 |
|  | XP\_065066428\_8507-model5.2 | -18.0675 |
|  | XP\_065066428\_8507-model5.3 | -13.8658 |
| # cluster 6 - Size: 1 |  |  |
|  | XP\_065066428\_8507-model6 | -21.6088 |
| # cluster 7 - Size: 2 |  |  |
|  | XP\_065066428\_8507-model7 | -19.26 |
|  | XP\_065066428\_8507-model7.1 | -18.9759 |
| # cluster 8 - Size: 1 |  |  |
|  | XP\_065066428\_8507-model8 | -18.2617 |
| # cluster 9 - Size: 1 |  |  |
|  | XP\_065066428\_8507-model9 | -18.0124 |
| # cluster 10 - Size: 1 |  |  |
|  | XP\_065066428\_8507-model10 | -17.7962 |
| # cluster 11 - Size: 2 |  |  |
|  | XP\_065066428\_8507-model11 | -16.5304 |
|  | XP\_065066428\_8507-model11.1 | -14.1703 |
| # cluster 12 - Size: 10 |  |  |
|  | XP\_065066428\_8507-model12 | -15.7698 |
|  | XP\_065066428\_8507-model12.1 | -14.9294 |
|  | XP\_065066428\_8507-model12.2 | -14.4962 |
|  | XP\_065066428\_8507-model12.3 | -14.3516 |
|  | XP\_065066428\_8507-model12.4 | -14.2766 |
|  | XP\_065066428\_8507-model12.5 | -14.2653 |
|  | XP\_065066428\_8507-model12.6 | -14.2358 |
|  | XP\_065066428\_8507-model12.7 | -13.8954 |
|  | XP\_065066428\_8507-model12.8 | -13.8544 |
|  | XP\_065066428\_8507-model12.9 | -13.6037 |
| # cluster 13 - Size: 8 |  |  |
|  | XP\_065066428\_8507-model13 | -15.7162 |
|  | XP\_065066428\_8507-model13.1 | -15.4641 |
|  | XP\_065066428\_8507-model13.2 | -15.2281 |
|  | XP\_065066428\_8507-model13.3 | -14.9303 |
|  | XP\_065066428\_8507-model13.4 | -14.8659 |
|  | XP\_065066428\_8507-model13.5 | -14.7244 |
|  | XP\_065066428\_8507-model13.6 | -14.0809 |
|  | XP\_065066428\_8507-model13.7 | -13.9676 |
| # cluster 14 - Size: 2 |  |  |
|  | XP\_065066428\_8507-model14 | -15.6561 |
|  | XP\_065066428\_8507-model14.1 | -15.228 |
| # cluster 15 - Size: 6 |  |  |
|  | XP\_065066428\_8507-model15 | -15.5654 |
|  | XP\_065066428\_8507-model15.1 | -14.6629 |
|  | XP\_065066428\_8507-model15.2 | -14.655 |
|  | XP\_065066428\_8507-model15.3 | -14.1548 |
|  | XP\_065066428\_8507-model15.4 | -14.1288 |
|  | XP\_065066428\_8507-model15.5 | -14.0409 |
| # cluster 16 - Size: 4 |  |  |
|  | XP\_065066428\_8507-model16 | -15.448 |
|  | XP\_065066428\_8507-model16.1 | -14.0002 |
|  | XP\_065066428\_8507-model16.2 | -13.9675 |
|  | XP\_065066428\_8507-model16.3 | -13.7549 |
| # cluster 17 - Size: 3 |  |  |
|  | XP\_065066428\_8507-model17 | -15.0601 |
|  | XP\_065066428\_8507-model17.1 | -13.8973 |
|  | XP\_065066428\_8507-model17.2 | -13.5687 |
| # cluster 18 - Size: 1 |  |  |
|  | XP\_065066428\_8507-model18 | -14.9892 |
| # cluster 19 - Size: 3 |  |  |
|  | XP\_065066428\_8507-model19 | -14.385 |
|  | XP\_065066428\_8507-model19.1 | -14.3817 |
|  | XP\_065066428\_8507-model19.2 | -14.3743 |
| # cluster 20 - Size: 28 |  |  |
|  | XP\_065066428\_8507-model20 | -14.3668 |
|  | XP\_065066428\_8507-model20.1 | -14.3528 |
|  | XP\_065066428\_8507-model20.2 | -14.3348 |
|  | XP\_065066428\_8507-model20.3 | -14.2842 |
|  | XP\_065066428\_8507-model20.4 | -14.2305 |
|  | XP\_065066428\_8507-model20.5 | -14.1516 |
|  | XP\_065066428\_8507-model20.6 | -14.1471 |
|  | XP\_065066428\_8507-model20.7 | -14.1454 |
|  | XP\_065066428\_8507-model20.8 | -14.1439 |
|  | XP\_065066428\_8507-model20.9 | -14.1331 |
|  | XP\_065066428\_8507-model20.10 | -14.0378 |
|  | XP\_065066428\_8507-model20.11 | -14.0378 |
|  | XP\_065066428\_8507-model20.12 | -14.003 |
|  | XP\_065066428\_8507-model20.13 | -13.9989 |
|  | XP\_065066428\_8507-model20.14 | -13.9343 |
|  | XP\_065066428\_8507-model20.15 | -13.9042 |
|  | XP\_065066428\_8507-model20.16 | -13.8871 |
|  | XP\_065066428\_8507-model20.17 | -13.8685 |
|  | XP\_065066428\_8507-model20.18 | -13.8121 |
|  | XP\_065066428\_8507-model20.19 | -13.8106 |
|  | XP\_065066428\_8507-model20.20 | -13.7969 |
|  | XP\_065066428\_8507-model20.21 | -13.788 |
|  | XP\_065066428\_8507-model20.22 | -13.7755 |
|  | XP\_065066428\_8507-model20.23 | -13.7707 |
|  | XP\_065066428\_8507-model20.24 | -13.6846 |
|  | XP\_065066428\_8507-model20.25 | -13.6363 |
|  | XP\_065066428\_8507-model20.26 | -13.543 |
|  | XP\_065066428\_8507-model20.27 | -13.5297 |
| # cluster 21 - Size: 2 |  |  |
|  | XP\_065066428\_8507-model21 | -14.1717 |
|  | XP\_065066428\_8507-model21.1 | -13.4662 |
| # cluster 22 - Size: 5 |  |  |
|  | XP\_065066428\_8507-model22 | -14.1307 |
|  | XP\_065066428\_8507-model22.1 | -14.0863 |
|  | XP\_065066428\_8507-model22.2 | -13.9571 |
|  | XP\_065066428\_8507-model22.3 | -13.7892 |
|  | XP\_065066428\_8507-model22.4 | -13.5618 |
| # cluster 23 - Size: 1 |  |  |
|  | XP\_065066428\_8507-model23 | -10.7156 |
|  |  |  |
